# Covalent immobilization of luminescent oxygen indicators reduces cytotoxicity

**DOI:** 10.1007/s10544-020-00495-3

**Published:** 2020-06-03

**Authors:** Hannu Välimäki, Tanja Hyvärinen, Joni Leivo, Haider Iftikhar, Mari Pekkanen-Mattila, Dhanesh Kattipparambil Rajan, Jarmo Verho, Joose Kreutzer, Tomi Ryynänen, Jonatan Pirhonen, Katriina Aalto-Setälä, Pasi Kallio, Susanna Narkilahti, Jukka Lekkala

**Affiliations:** 1 Faculty of Medicine and Health Technology, Tampere University, Korkeakoulunkatu 3, 33720 Tampere, Finland; 2 Faculty of Medicine and Health Technology, Tampere University, Arvo Ylpön katu 34, 33520 Tampere, Finland

**Keywords:** Luminescent-based oxygen sensing, PtTFPP cytotoxicity, Covalently immobilized indicators, Human induced pluripotent stem cell-derived cells, hiPSC-derived neurons and cardiomyocytes

## Abstract

Luminescence-based oxygen sensing is a widely used tool in cell culture applications. In a typical configuration, the luminescent oxygen indicators are embedded in a solid, oxygen-permeable matrix in contact with the culture medium. However, in sensitive cell cultures even minimal leaching of the potentially cytotoxic indicators can become an issue. One way to prevent the leaching is to immobilize the indicators covalently into the supporting matrix. In this paper, we report on a method where platinum(II)-5,10,15,20-tetrakis-(2,3,4,5,6-pentafluorphenyl)-porphyrin (PtTFPP) oxygen indicators are covalently immobilized into a polymer matrix consisting of polystyrene and poly(pentafluorostyrene). We study how the covalent immobilization influences the sensing material’s cytotoxicity to human induced pluripotent stem cell-derived (hiPSC-derived) neurons and cardiomyocytes (CMs) through 7–13 days culturing experiments and various viability analyses. Furthermore, we study the effect of the covalent immobilization on the indicator leaching and the oxygen sensing properties of the material. In addition, we demonstrate the use of the covalently linked oxygen sensing material in real time oxygen tension monitoring in functional hypoxia studies of the hiPSC-derived CMs. The results show that the covalently immobilized indicators substantially reduce indicator leaching and the cytotoxicity of the oxygen sensing material, while the influence on the oxygen sensing properties remains small or nonexistent.

**Electronic supplementary material:**

The online version of this article (10.1007/s10544-020-00495-3) contains supplementary material, which is available to authorized users.

## Introduction

Molecular oxygen has a large impact on the viability and functioning of cells and tissues, thus requiring careful monitoring and control in cell cultures (Wilson 2008; Bunn and Poyton 1996). Today, a widely applied technique for oxygen monitoring is based on the use of luminescent indicators, which is replacing Clark’s electrodes in many fields (Wolfbeis 2015). The luminescence-based technique is sensitive, and it provides a large set of sensing schemes and modalities, including sensing spots, fiber-optic set-ups, planar films, smart scaffolds as well sensing beads for 3D oxygen imaging (Wang and Wolfbeis 2014). In addition, the sensitivity and measurement range can be tuned with the appropriate choice of indicators and matrix materials (Quaranta et al. 2012; Mills 1997). Moreover, the luminescent technique does not consume oxygen and integrates well into many systems, which are especially important features in microfluidic or miniaturized cell cultures (Papkovsky and Dmitriev 2013; Grist et al. 2010; Pfeiffer and Nagl 2015; Abaci et al. 2012).

The luminescent oxygen indicators are typically embedded in a solid, oxygen-permeable matrix, most often in a suitable polymer in cell culture applications. However, when monitoring sensitive cultures, it is important that the indicators do not leach out of the matrix, as they can be cytotoxic and hamper the cells even in minute concentrations. In addition, the leached indicators may prove fatal through the phototoxic effects – the effect that is utilized in photodynamic cancer therapy (Agostinis et al. 2011).

The indicator leaching can be mitigated in a few ways. Firstly, the sensing material can be covered with an indicator-free but oxygen-permeable shielding layer. This approach adds to the complexity of the system but can be effective against both the cytotoxic and phototoxic effects (Thomas et al. 2009). Ideally, the shielding layer can also have a favorable effect on the hydrophilicity of the sensing material and make it more attractive to cells (Xue et al. 2014). On the other hand, a separate layer may create new adhesion problems and, due to the increased diffusion distance, increase the response time as well. Moreover, the layer can generate calibration problems due to the diffusion driven migration of the indicators (O’Riordan et al. 2005).

Another option is to immobilize covalently the indicators into the matrix. This technique requires both suitable indicators and matrix constituents with carefully chosen chemical functionalities. Porphyrins with pentafluorophenyl groups, such as platinum(II)-5,10,15,20-tetrakis-(2,3,4,5,6-pentafluorphenyl)-porphyrin (PtTFPP), are excellent in this respect, and they have been covalently immobilized in many matrices, including polystyrene (PS), poly(2-hydroxyethyl methacrylate)-co-polyacrylamide (PHEMA) (Tian et al. 2010a; Wu et al. 2018), silica gel (Tian et al. 2010b), poly(styrene-co-pentafluorostyrene) and organically modified silica (Ormosil) (Koren et al. 2012). In addition, nanoparticles containing covalently immobilized metalloporphyrins and conjugated polymer antennas and have been prepared for oxygen imaging in cells and 3D tissue models (Dmitriev et al. 2015; Qiao et al. 2019).

Our group has recently developed a modular platform for cell culturing (Rajan et al. 2018). The platform incorporates numerous sensing functionalities, including microelectrode arrays (MEAs), optical microscopy and luminescence-based oxygen sensing, and it has been applied for temperature (Mäki et al. 2018) and hypoxia (Välimäki et al. 2017) studies of human induced pluripotent stem cell-derived (hiPSC-derived) cardiomyocytes (CMs). In the hypoxia studies, we used Pt(II) octaethyl-porphyrinketone (PtOEPK) as physically embedded in a PS matrix for the oxygen monitoring, as reports exist on the compatibility of such a sensing material with mammalian cells (Sinkala and Eddington 2010; Oppegard and Eddington 2013). In our studies, however, it became evident that the physically embedded PtOEPK in PS is not sufficiently compatible with hiPSC-derived cells, at least for longer culturing times. Therefore, the above mentioned reports of PS with covalently immobilized porphyrins evoked our interest, even though these reports typically either lacked cytotoxicity tests (Koren et al. 2012), or the tests covered only relatively short (24 h) culturing of bacteria (Tian et al. 2010b) or cancer cells (Tian et al. 2010a).

This paper studies the effect of the covalently immobilized indicators on the cytotoxicity of the luminescent oxygen sensing material. The study focuses on materials based on PtTFPP in PS and on their compatibility with hiPSC-derived neurons and CMs. For the covalent immobilization of PtTFPP, we utilize the thiol-mediated method (Koren et al. 2012) with some modifications. Briefly, a polymer blend of PS and poly(pentafluorostyrene) (PPFS) is created, and PtTFPP is covalently linked to PPFS. PtOEPK cannot be immobilized with the method, but a physically embedded PtOEPK-PS reference material is included in the tests because of its popularity (Quaranta et al. 2012; Grist et al. 2010). After presenting the methods, we characterize the manufactured sensing materials in terms of their oxygen sensing properties. Then we assess the cytotoxicity by culturing hiPSC-derived neurons and CMs in close contact with individual materials for 7–13 days and compare the viability of the cultures. In addition, we demonstrate how the material with covalently immobilized indicators is successfully applied to oxygen sensing when monitoring the hypoxia-induced behavioral changes of the hiPSC-derived CMs. We complete the study by analyzing how the covalent immobilization influences the indicator leaching.

## Materials and methods

The oxygen indicators PtTFPP and PtOEPK were purchased from Livchem Logistics. The PS pellets, PPFS powder and the solvents were from Sigma.

### Preparation of the polymer blend with covalently immobilized indicators

**Fig. 1:**
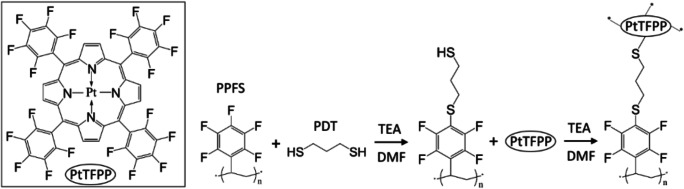
The chemical scheme of the thiol-mediated covalent immobilization of PtTFPP

Most of the preparation steps were adapted from Koren et al. (2012). Briefly, 1.00 g of PS and 6.3 mg of PPFS were dissolved in 20 ml of dimethylformamide (DMF) at 60 °C. Then 300 μl of 1,3-propanedithiol (PDT) cross-linker and 500 μl of triethylamine (TEA) were added to the solution at 75 °C. The solution was stirred at 75 °C for six hours. The precipitates were washed with methanol (MeOH) and ethanol (EtOH) and dried at 60 °C for 18 h. Next, 500 mg of the precipitate was dissolved in 20 ml of DMF and stirred at 75 °C overnight. In a separate vessel, 50 mg of PtTFPP was dissolved in 25 ml of DMF and 250 μl of TEA at 75 °C, and then added into the polymer blend solution. After six hours at 75 °C, the solution was washed with MeOH and EtOH to remove excess TEA and unbound PtTFPP. The resulting precipitate, a blend of PS and PPFS with covalently immobilized PtTFPP, was dried on a hot plate at 60 °C overnight. Finally, the precipitate was dissolved in toluene to produce a 4.0% *w*/w solution for the manufacturing of the sensing films and spots. The PtTFPP concentration of the prepared material, denoted as Cov1, was estimated with optical absorbance measurements. Typically, the absorbance spectrum of Cov1 in toluene was similar to that of the reference material containing 0.05% *w*/w of physically embedded PtTFPP (see Fig. S1 in Online Resource 1). Figure 1 shows the chemical scheme of the covalent immobilization of PtTFPP.

### Preparation of the reference polymers with physically embedded indicators

Reference sensing materials with physically embedded indicators were prepared for comparison. Briefly, PS pellets were dissolved in toluene (4.0% *w*/w), and PtTFPP was added into the solution in two concentrations: Material Ref1 contained 0.05% w/w (same as Cov1) and Ref2 0.5% w/w of PtTFPP with respect to PS. The reference material with the higher indicator concentration was expected to perform more unsatisfactorily in the cytotoxicity tests. Finally, a PS solution with physically embedded PtOEPK (0.05% w/w) was prepared as an additional reference material (Ref3). Table 1 Lists all the prepared oxygen sensing materials

**Table 1:** The oxygen sensing materials used in the study

Code	polymer	Indicator	Type	Indicator concentration [% w/w]
Cov1	PS-PPFS blend	PtTFPP	Covalently immobilized	0.05^1^
Ref1	PS	PtTFPP	Physically embedded	0.05
Ref2	PS	PtTFPP	Physically embedded	0.5
Ref3^2^	PS	PtOEPK	Physically embedded	0.05

^1^An estimate based on absorption measurements

^2^Used only as an additional reference in the cytotoxicity tests

### Preparation of the oxygen sensing films and spots

Glass plates (49 mm × 49 mm × 1 mm; *n* = 1.5171; Gerhard Menzel GmbH) were sonicated in acetone and isopropanol, rinsed with deionized (DI) water and dried with nitrogen. The plates were treated with oxygen plasma for five minutes and pre-coated with hexamethyldisilazane (HMDS) in a vacuum at 150 °C (Hotplate HMDS-OPTIhot SVT20). Oxygen sensing films were manufactured by spin coating the prepared polymer solutions with a speed of 3000 rpm, resulting in a film thickness of approximately 500 nm (Hall et al. 1998; Välimäki et al. 2017). The plates were placed on a hot plate at 100 °C for ten minutes, and then left to dry for 12 h.

Alternatively, oxygen sensing spots were manufactured by pipetting 0.2–0.3 μL drops of the polymer solution with a small glass capillary on the sonicated and pre-coated glass plates and letting the spots dry in cleanroom conditions for 12 h. The resulting sensor spots had a diameter of between 1.5 mm and 2.0 mm and a thickness of between 3 μm and 10 μm at the center.

### Luminescence spectroscopy

The absorption spectra of the individual sensing films were measured with an Ocean Optics UV-VIS spectrometer (Jaz Series). The emission spectra were measured with Fluorolog Yobin Yvon-SPEX. The excitation wavelength was set to 390 nm with a 5 nm slit. The recording took place between 600 and 750 nm with a 1 nm slit, and the spectra were corrected for the wavelength dependent sensitivity of the photomultiplier tube.

### Oxygen sensing

**Fig. 2:**
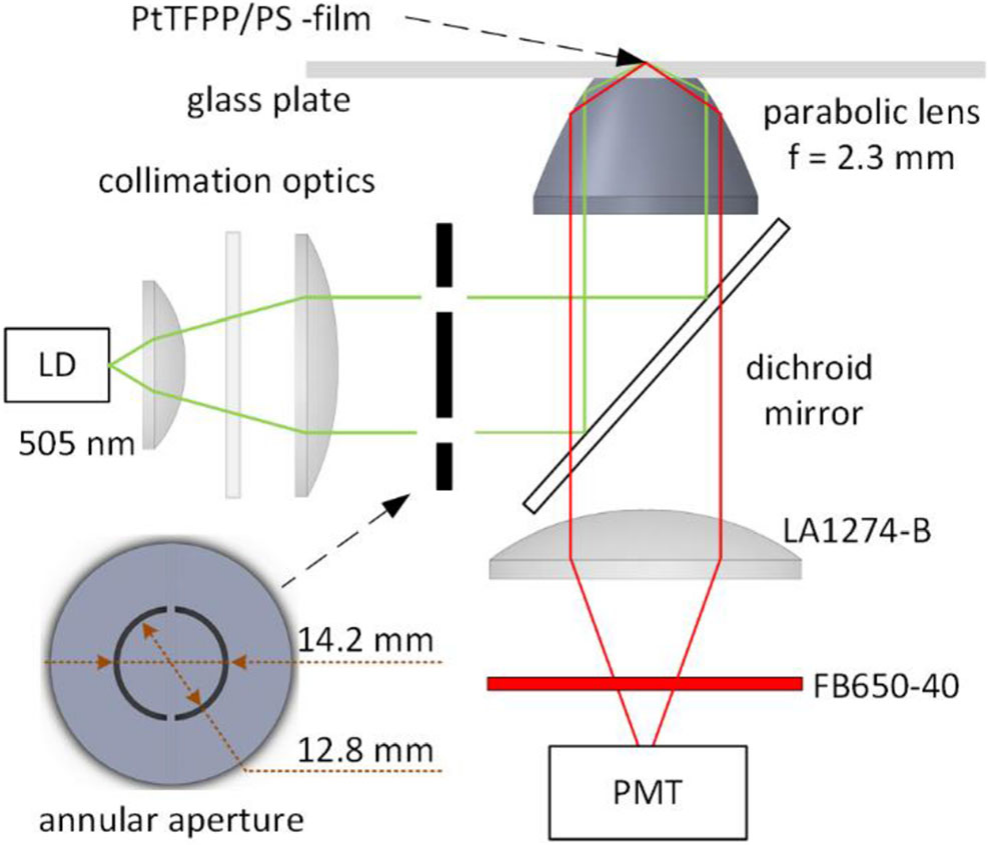
The optical set-up used in the oxygen measurements. The parabolic lens is in an optical contact with a glass plate, spin coated with a PtTFPP/PS film. The output of the excitation laser diode (LD, 505 nm) is collimated, attenuated and directed through a customized annular aperture, a dichroic mirror and the parabolic surface to the focal point of the system, which is located on the top surface of the glass plate. The luminescence emission is collected with the parabolic lens, a plano-convex lens and filtered through a bandpass filter to the photo multiplier tube (PMT)

The optical set-up reported earlier (Välimäki et al. 2017) was applied with some modifications (Fig. 2). The glass plates with spin coated PtTFFP/PS films were positioned on a parabolic lens (*n* = 1.515; f = 2.3 mm; bottom diameter 25.00 mm; top diameter 11.18 mm; custom manufactured by Shanghai Optics). The optical contact between the glass plate and the parabolic was ensured with immersion oil. The excitation laser diode (LD, Single Mode Sharp GH05035A2G, 505 nm, 35 mW) was from BeamQ. The output of the laser was collimated, attenuated with a neutral density filter (NE10A) and directed through an annular aperture defined by the diameters of 12.8 mm and 14.2 mm (3D printed out of photoactive resin, Formlabs, Form 2). The beam was reflected by a dichroic mirror (DMLP550R) to the parabolic surface that focused the beam into the focal point, located on the optical axis on the top surface of the glass plate. The chosen parameters result in a range of excitation angles between 65.9° and 71.4°, which is notably larger than the critical angle between glass and water (61.4°). The luminescence emission was collected with the parabolic lens and a plano-convex lens (LA1274-B, f = 40 mm) and filtered through a bandpass filter (FB650–50). All the filters and standard lenses were from Thorlabs.

The luminescence lifetime was determined by quadrature synchronous detection. The excitation laser diode was at 2 kHz, and the emission was detected with a photomultiplier tube (PMT, Hamamatsu H12056) equipped with a tailored transimpedance amplifier. The synchronous detector had a bandwidth of 23 Hz and the I/Q outputs were sampled to produce phase angle measurements at a data rate of 10 Hz. The excitation signal was used as a phase reference.

Tailored polydimethylsiloxane (PDMS) 1-well culture chambers with surrounding gas-control structures were utilized as calibration chambers (Välimäki et al. 2017). The chambers were mounted directly on the oxygen sensing films.

### Photostability measurements

To assess the photostability of the prepared oxygen sensing films, a UV curing spotlight device (Dymax BlueWave 50 AS) was applied. The fiber output of the device (beam diameter 5.0 mm, 3 Wcm^−2^ at 320 nm – 440 nm) was directed to the sensing films at 1.0 mm distance. Short exposures followed with luminescence measurements were carried out in controlled oxygen conditions.

### Indicator leaching tests

The indicator leaching from individual sensing materials was assessed with a short acetone treatment. Glass plates with spin-coated oxygen sensing films were held upright and immersed halfway in acetone for 20 s. The calibration chambers were mounted on the plates in such a way that the oxygen sensing responses could be recorded both on the acetone-treated and the non-treated areas. The treatment-induced attenuation in the luminescence emission and the respective changes in the oxygen sensing capability were used to assess the ability of individual sensing materials to limit the indicator leaching.

### hiPSC-derived neurons

#### Differentiation

**\**Neuronal cells were derived from the hiPSC-line 10,212.EURCCs. The hiPSCs were maintained on top of a human feeder cell layer according to Rajala et al. (2007), and before differentiation expanded in a feeder-free culture (Hongisto et al. 2017). The cortical neural differentiation was based on (Shi et al. 2012) with some modifications. Neuronal cells were differentiated in a neural maintenance medium consisting of 1:1 D-MEM/F12 with Glutamax and Neurobasal, 0.5% N2, 1% B27 with Retinoic Acid, 0.5 mM GlutaMAX, 0.5% non-essential amino acids (NEEA), 50 μM 2-mercaptoethanol (all from Thermo Fisher Scientific), 2.5 μg/ml insulin (Sigma) and 0.1% penicillin/streptomycin (Thermo Fisher Scientific). For neural induction, the culture dishes were coated with Matrigel (Corning) and neural maintenance medium supplemented with 100 nM LDN193189 and 10 μM SB431542 (both from Sigma) for 12 days. After neural induction, the culture dishes were coated with 100 μg/ml poly-L-ornithine (PO, Sigma) in borate buffer and 15 μg/ml mouse laminin (Sigma) in phosphate buffered saline (PBS). From day 13 to day 25, the neural progenitor cells were expanded in the neural maintenance medium supplemented with 20 ng/ml of fibroblast growth factor-2 (FGF2) (R&D Systems). From day 26 onwards, the neural maintenance medium was supplemented with 20 ng/ml of brain-derived neurotrophic factor (BDNF, R&D Systems), 10 ng/ml glial-derived neurotrophic factor (GDNF, R&D Systems), 500 μM dibutyryl-cyclicAMP (db-cAMP, Sigma) and 200 μM ascorbic acid (Sigma) for neuronal maturation.

#### Cytotoxicity tests

The oxygen sensing films were sterilized using 70% ethanol. Custom-made PDMS rings were bonded to the oxygen sensing films in order to create six separate cell culture areas (Kreutzer et al. 2012). These cell culture areas were coated with 100 μM/ml PO for one hour at 37 °C and washed three times with sterile H_2_O and left to dry at room temperature. Double coating was finished off with 50 μg/ml mouse laminin overnight at 4 °C. The cells were plated at a density of 290,000/cm^2^ on day 32 of the differentiation. The mediums were refreshed three times a week, and the cultures were followed for seven days before the cell characterizations.

The viability of the neurons on D7 was studied using a LIVE/DEAD® Viability/Cytotoxicity Kit for mammalian cells (Thermo Fisher Scientific) according to Ylä-Outinen et al. (2014). Briefly, the cultures were incubated for 30 min in a dye solution containing 0.1 μM calcein-AM for the detection of the live cells and 0.5 μM ethidium homodimer-1 live for the detection of the dead cells. The samples were imaged with an Olympus IX51 microscope. The CellProfiler software (Carpenter et al. 2006) was used to quantify the area covered by the cells.

The number of the cells and the percentage of neurons were evaluated with immunocytochemical staining on D7 according to Lappalainen et al. (2010). The nuclei were stained with 4′,6-diamidino-2-phenylindole (DAPI), and neurons with a primary antibody β-tubulin_III_ (1:500; T8660, Sigma) and secondary antibody Alexa Fluor 488 (1:400; A21202, Thermo-Fisher). Imaging as above, and the number of the nuclei and the percentage of the β-tubulin-positive cells were determined with the CellProfiler and CellProfiler Analyst (Jones et al. 2008) software.

### hiPSC-derived CMs

#### Differentiation

CMs were differentiated from the hiPSC-line UTA.04602.WT (Lahti et al. 2012). The differentiation was carried out by co-culturing hiPSCs with mouse visceral endodermal-like (END-2) cells (Mummery et al. 2012). The beating hiPSC-CM clusters were mechanically excised from the differentiation cultures 20–30 days after the differentiation initiation.

#### Cytotoxicity test

PDMS rings, similar to those used with neuronal cells, were bonded to the glass plates. An oxygen sensing spot with a diameter of between 1.5 and 2.0 mm was manufactured onto the center of each cell culture area (diameter of 3.0 mm). The structures were sterilized with 70% ethanol. The cell culture areas were hydrophilized with fetal bovine serum (FBS, from Lonza) for five minutes and coated with 0.1% gelatin type A (Sigma) for one hour at room temperature. For each well, 1–3 beating hiPSC-CM clusters were plated. The CMs were plated and cultured in KnockOut Dulbecco’s Modified Eagle Medium (KO-DMEM) (Lonza) with 20% FBS, 1% NEAA (Cambrex), 2 mM Glutamax (Invitrogen) and 50 U/ml penicillin/streptomycin (Lonza). The medium was refreshed twice a week.

The viability of the CM clusters was assessed with phase microscopy on D7 and D13. The clusters in each well were evaluated in terms of the attachment and beating. If the CM cluster in the well was both well-attached and beating, the cluster was given a score of 2. If the cluster was neither well-attached nor beating, it was given a score of 0. The well-attached but non-beating, as well as the poorly attached but beating clusters, were evaluated as a score of 1.

#### Monitoring pO_2_ in hiPSC-derived CM cultures

A glass substrate with a customized single-cell MEA structure was used (Ryynänen et al. 2018). The MEA structure contained an electrode-free area, where the oxygen sensing spot with a diameter of approximately 1.5 mm was manually manufactured. A PDMS 1-well culture chamber (Välimäki et al. 2017) was bonded to the substrate. Cell plating was as described above, and the cultures were placed in a standard incubator for five days for initial stabilization. The medium was refreshed after four days. After the initial stabilization, the culture was moved to the modular system (Rajan et al. 2018), where the temperature was stabilized at T = 37 °C and the gas content of the culture was controlled. The baseline conditions (O_2_ 10%, CO_2_ 5%, N_2_ 85%) continued for 13 h until the hypoxic conditions (O_2_ 1%, CO_2_ 5%, N_2_ 94%) were set. After 8 h, the baseline conditions were restored. The functional responses to the changing oxygen conditions were followed by recording simultaneous MEA signals and microscopy videos. As a detailed analysis of the signals is out of the scope of this paper, the functional changes of the CM clusters are reported only in terms of video-based beating frequency.

### Statistical tests

The non-parametric Mann-Whitney U-test (two-tailed) was applied in the statistical analysis. A value of *p* < 0.05 was considered as statistically significant. All the statistical tests were performed with SPSS Statistics software v25.0.

## Results and discussion

Various experimental set-ups were used to study the effect of the covalently immobilized indicators on the properties of the sensing material in terms of the oxygen sensing performance, cytotoxicity to hiPSC-derived neurons and CMs and ability to limit the indicator leaching.

### Oxygen sensing properties

#### Spectral properties

The covalent immobilization of the indicators had no significant effects on the spectral properties of the sensing material. The absorption maximum was located at 397 nm and the emission maximum at 650 nm for both the covalently immobilized (Cov1) and physically embedded PtTFPP (Ref1, Ref2) samples. The spectra are shown in Fig. S2 in Online Resource 1.

#### Oxygen sensing characteristics

**Fig. 3:**
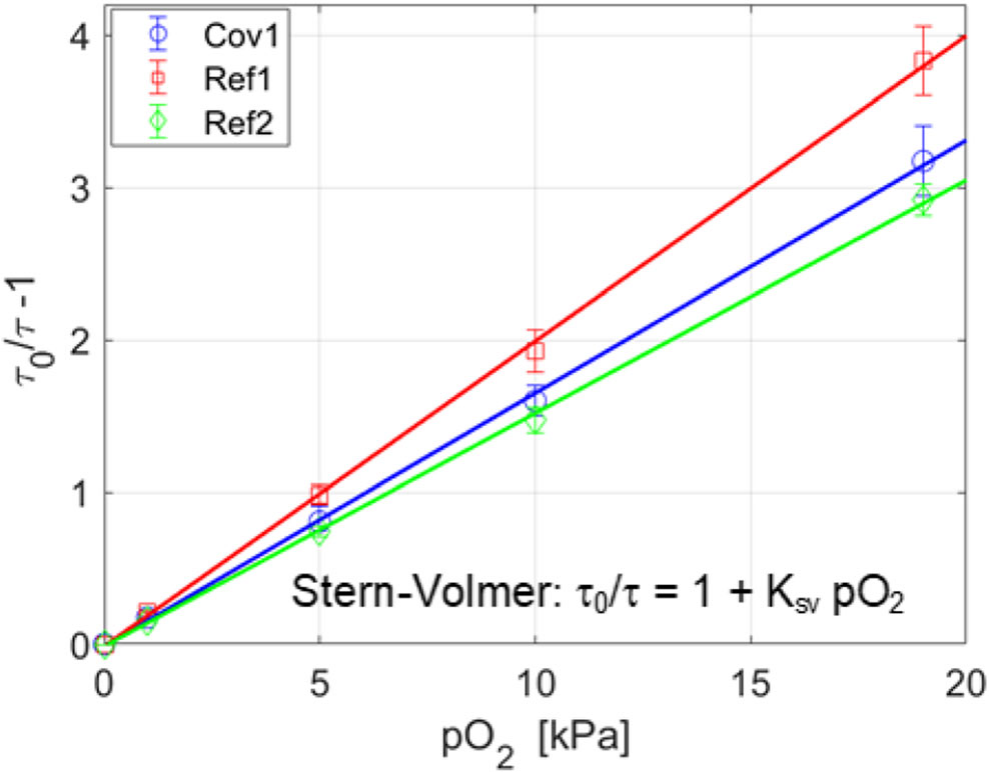
Stern-Volmer plots of the studied PtTFPP-based oxygen sensing films

A calibration chamber was mounted on the oxygen sensing films. The oxygen concentration of the chamber was controlled, and the sensor amplitude and phase responses were recorded. The corresponding luminescence lifetime-type Stern-Volmer plots (Lakowicz 2006) are shown for the PtTFPP-based films in Fig. 3. The plots are nearly linear in all three cases, suggesting the dominance of a single luminescence lifetime. The values for the Stern-Volmer constant K_sv_ (i.e. the slope of the plot) are in the same range, and the relatively small differences in the typical values stem from the differences in oxygen diffusion constants, unquenched lifetimes and quantum yields ─ all affected by the indicator concentration and the polymer composition. Table 2 summarizes the oxygen sensing characteristics of the sensing films. Overall, the covalent immobilization of the indicators has no major influence on the oxygen sensing characteristics. For completeness, the table also includes typical values for the PtOEPK-based films.

**Table 2:** The oxygen sensing characteristics of the studied sensing films**:** absorption maxima (abs λ_max_); emission maxima (em λ_max_); luminescence lifetimes (τ_0_); Stern-Volmer constants (K_sv_) and root mean square (RMS) noise values

Code	abs λ_max_ [nm]	em λ_max_ [nm]	τ_0_^1^ [μs]	K_sv_ [kPa^−1^]	RMS noise [°]^2^
Cov1	397, 509, 542	650	61	0.166	0.031
Ref1	397, 509, 542	650	64	0.198	0.025
Ref2	397, 509, 542	650	58	0.151	0.010
Ref3	398, 592^3^	759^3^	61^3^	0.235^4^	0.028^4^

^1^estimated with a fitted monoexponential decay curve at pO_2_ = 0.0 kPa;

^2^At 5.0 kPa; ^3^values from (Papkovsky et al. 1995);

^4^measured with the setup as in (Välimäki et al. 2017)

#### Photostability

**Fig. 4:**
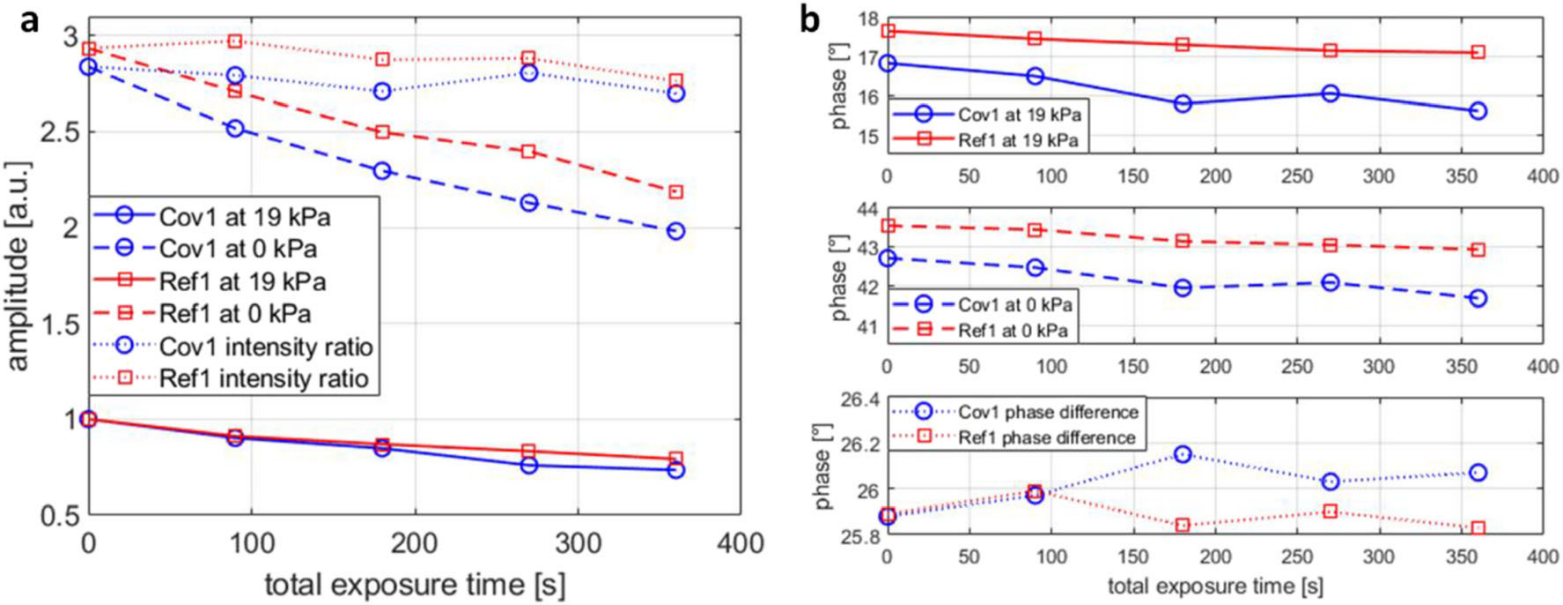
Changes in the oxygen sensor amplitude (**a**) and phase (**b**) responses with sensing films Cov1 and Ref1, when repeatedly exposed to 90 s of UV radiation with irradiance level of 3000 mWcm^−2^. The values at pO_2_ = 19 kPa are shown as solid lines; at pO_2_ = 0 kPa as dashed lines; the ratio of the amplitude responses (**a**) and the difference of phase responses (**b**), at pO_2_ = 19 kPa and at pO_2_ = 0 kPa, are shown as dotted lines

The photostability of the sensing materials Cov1 and Ref1 was compared. As PtTFPP is known for its excellent photostability properties (Amao 2003), we utilized a high-power UV curing spotlight device and recorded the sensor responses after a sequence of powerful UV exposures. Figure 4a shows typical amplitude responses for the films Cov1 and Ref1 at pO_2_ = 19 kPa (solid lines) and pO_2_ = 0 kPa (dashed lines), as a function of the cumulative UV exposure time. The graphs are normalized with the initial intensity at pO_2_ = 19 kPa. Even under the strong UV exposure, both films showed firm intensity stability. At the end of the tests, both films had lost approximately 30% of their emission power, but nevertheless, the ratio of intensities at pO_2_ = 0 kPa and pO_2_ = 19 kPa remained nearly the same (dotted lines). The corresponding phase responses are shown in Fig. 4b. The cumulative UV exposure resulted in a low negative phase change for both films, thus indicating a minor shortening of the luminescence lifetimes, and the effect was slightly more pronounced for the film Cov1 than for Ref1. However, the differences between the films were small, and it is evident that the covalent immobilization of PtTFPP generates no serious effects on the oxygen sensing performance in terms of sensitivity, noise level or photostability.

### Cytotoxicity to hiPSC-derived cells

#### Neurons

**Fig. 5:**
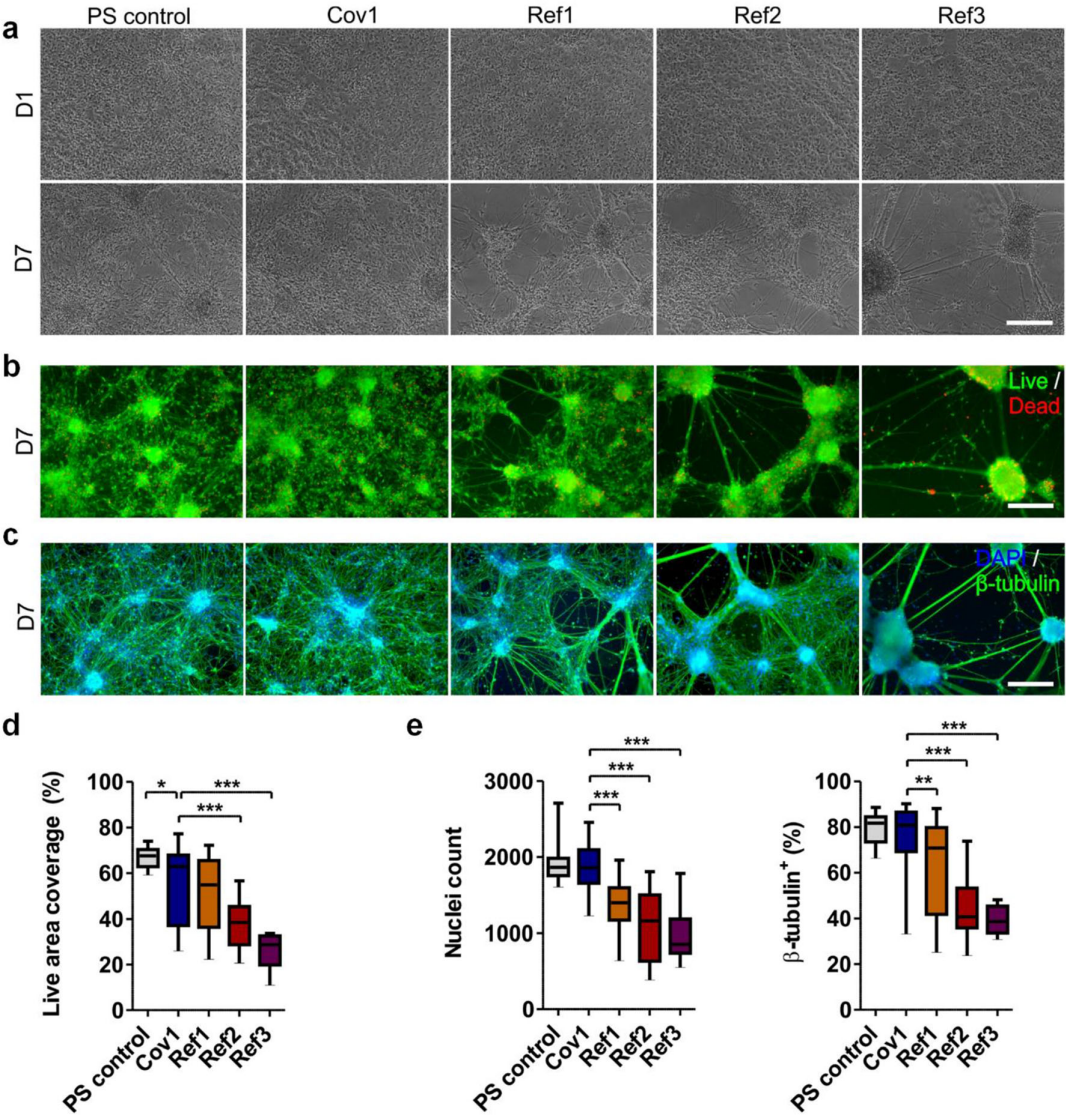
The viability and immunocytochemical characterization of the hiPSC-derived neuronal cells cultured on different oxygen sensing films and a plain PS control film. (**a**) Representative phase contrast images after one (D1) and seven days (D7) in culture. (**b**) The Live/dead staining at D7. (**c**) The immunocytochemical staining of cell nuclei (DAPI) and neuronal cells (β-tubulin) at D7. The scale bar represents 200 μm in all images. (**d**) The areal coverage of live cells. (**e**) The number of DAPI-positive cell nuclei and the percentage of β-tubulin-positive neurons. For all quantifications, 9 ≤ *n* ≤ 37, derived from 1 to 2 experiments (Table S1 in Online Resource 1) and the data is presented as median and interquartile range with whiskers showing the minimum and maximum values. Statistical analysis according to Mann-Whitney U-test (two-tailed). Statistical significances are denoted as * *p* < 0.05; ** p < 0.01; *** p < 0.001

The hiPSC-derived neurons were plated on individual sensing films and followed for up to seven days in an incubator. In addition to the films listed in Table 1, a film containing only PS (PS control) was used as a basic reference in the cytotoxicity tests. First, the morphology and spreading of the neuronal cells were evaluated with phase contrast images. One day (D1) after the cell plating, all groups showed equal cell attachment, spreading and neuronal morphology (Fig. 5a). After seven days (D7), both the PS control and Cov1 still supported neuronal growth. However, on the films Ref1, Ref2 and Ref3 the cells started to retract into cell aggregates (Fig. 5a).

Live-dead staining was performed on D7 to assess the viability and spreading of the neuronal cells. As with the phase contrast images, some live-dead images showed similar retraction into cell aggregates as on the films Ref1, Ref2 and Ref3 (Fig. 5b). Very few dead cells were spread among the living cells (Fig. 5b). We quantified the coverage of the live cells in the image area (Fig. 5d). There was a small but statistically significant difference between Cov1 and PS control (medians 63% and 68%, *p* < 0.05). On Ref1, the live cell coverage was lower than for Cov1 (median 55%), but not significantly so. However, the live cell coverages on Ref2 and Ref3 were significantly lower than they were on Cov1 (medians 38% and 29%, *p* < 0.001).

Immunocytochemical staining was performed on D7 to quantify the total cell count and the cells positive for the neuronal marker β-tubulin (Fig. 5c). A similar nuclei count (DAPI staining) was observed for the PS control and Cov1 films (medians 1862 and 1945, Fig. 5e). However, the films Ref1, Ref2 and Ref3 did not support cell growth as well as Cov1 (medians 1397, 1163 and 853, in all *p* < 0.001). On the PS control and Cov1, over 80% of cell population expressed the β-tubulin neuronal marker (Fig. 5e). However, on Ref1 the percentage of neurons was significantly lower (median 71%, *p* < 0.01), and both Ref2 and Ref3 showed only poor support for the neuronal identity (medians 41% and 39%, p < 0.001).

In conclusion, the hiPSC-derived neuronal cells attached, spread and sustained their neuronal identity most efficiently on the film Cov1 containing covalently immobilized PtTFPP. The reference film with equal but physically embedded concentration of PtTFPP (Ref1) lowered the spreading of the live cells, decreased the total number of the attached cells, and failed to support the neuronal phenotype as effectively. Furthermore, increasing the concentration of the physically embedded PtTFPP (Ref2) intensified all negative effects and, on the other hand, the removal of all physically embedded PtTFPP (PS control) removed all negative effects. Therefore, the results strongly suggest the cytotoxicity of PtTFPP to hiPSC-derived neurons. However, the most pronounced cytotoxic effects were created by the sensing material containing physically embedded PtOEPK (Ref3).

**CMs.** In the cytotoxicity tests with hiPSC-derived CMs, we used oxygen sensing spots instead of spin-coated sensing films. In these tests, however, the number of the samples, were substantially lower than in the tests with neurons and, in addition, the tests were partly hampered by unexpected long-term attachment issues of the sensing spots, which necessitated the merging of the reference data sets in the statistical analysis. Therefore, the cytotoxicity results with the hiPSC-derived are not directly comparable with the toxicity results with neurons (test details are shown in the Online Resource 1). However, the test results nevertheless suggest that the material with the covalently immobilized PtTFPP (Cov1) displays reduced cytotoxicity (*p* < 0.05), compared to the combined set of reference materials with physically embedded PtTFPP (Ref1) or PtOEPK (Ref3) (Fig. S3 in Online Resource 1).

### Application example: Monitoring pO2 in hiPSC-derived CM cultures

In order to study only the direct cytotoxicity-related effects of individual sensing materials, the cytotoxicity tests were carried out in the dark in the incubator. In real applications, however, the photoexcitation of the indicator can generate, as an unwanted byproduct, a certain amount of singlet oxygen ^1^O_2_. Exposure to ^1^O_2_ can be irreversibly damaging or even lethally toxic to cells (Dahl 1993). Fortunately, the lifetime (τ ≈ 3.5 μs) and mean diffusion distance (of order 200 nm for 5τ) of ^1^O_2_ is very limited in aqueous solutions (Ogilby 2010). Thus, to do any damage, ^1^O_2_ should be produced inside, or at least very close to the cell membrane. Therefore, preventing the indicator leaching, applying low excitation power and preferably avoiding direct illumination of the cells should generally strongly mitigate against phototoxic issues in oxygen sensing.

To demonstrate the use of the developed non-cytotoxic oxygen sensing material in actual cell culture monitoring, we applied oxygen sensing material Cov1 together with the reported sensing set-up (Fig. 2) in the hypoxia studies of hiPSC-derived CMs. The hiPSC-derived CM clusters are exceptionally well-suited for such studies and demonstrations, as evident changes in their beating characteristics can be expected when the extracellular oxygen tension changes. Such changes can be straightforwardly analyzed with video microscopy or MEA recordings.

**Fig. 6:**
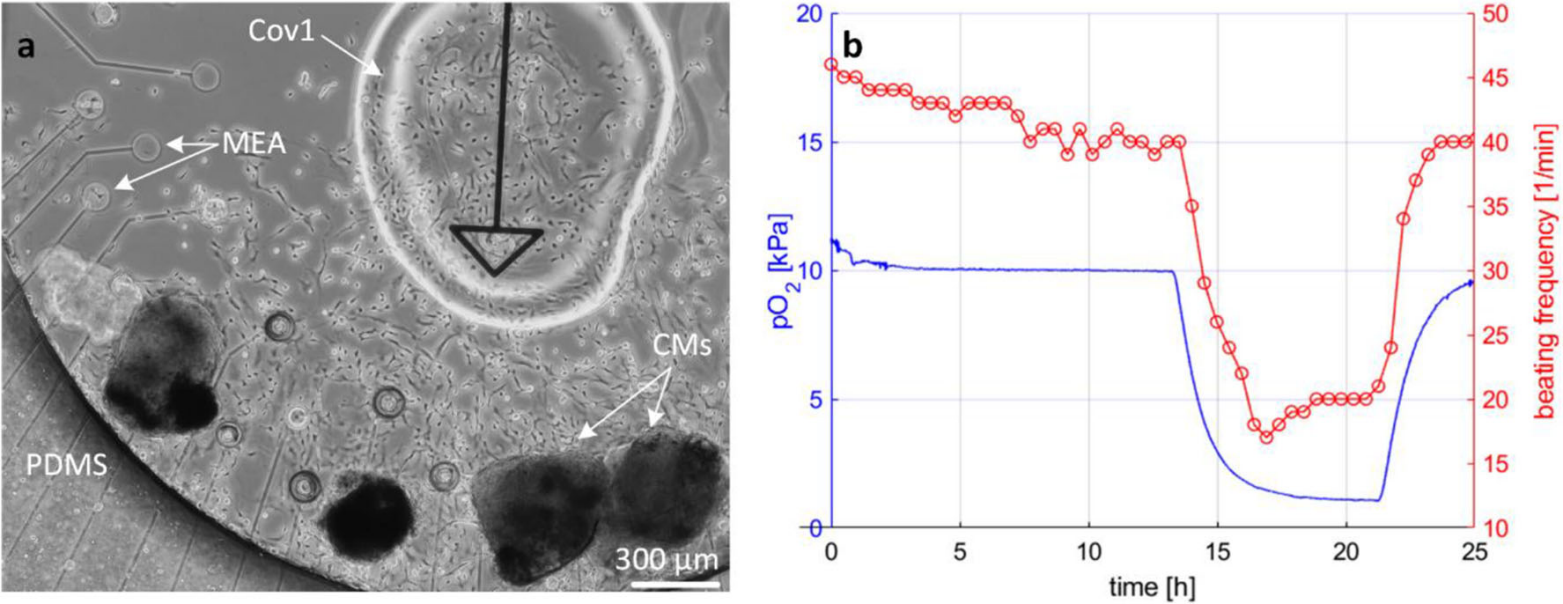
(**a**) A phase contrast image of four hiPSC-derived cardiomyocyte clusters during hypoxia studies. (**b**) The measured oxygen partial pressure and video-based beating frequency analysis of one cluster

The clusters were cultured on a customized MEA substrate (Ryynänen et al. 2018), and the environmental conditions were monitored and controlled by the modular cell-culturing system (Rajan et al. 2018). Figure 6a displays a phase contrast image of four CM clusters and an oxygen sensing spot on the MEA substrate after five days in vitro. The morphology and large spread of the cell clearly indicates the non-cytotoxicity of the material Cov1. The oxygen sensor response was recorded once a minute with a three second illumination time. The measured oxygen partial pressure and the beating frequency for one of the clusters for the first 25 h are shown in Fig. 6b. The beating frequencies were calculated from the recorded videos according to methods described by Ahola et al. (2014), and they display a clear correlation with the measured oxygen partial pressure. During numerous similar experiments, the oxygen measurements have not been observed to have any adverse effects on the hiPSC-derived CM clusters.

### Indicator leaching tests

The cytotoxicity test results are presumably directly related to the extent that the polymer matrix can limit the indicator leaching. However, with the tested materials, it is challenging to directly assess the leaching in the aqueous solutions used in cell culturing. Indeed, the leaching of physically embedded PtTFPP and PtOEPK out of polystyrene in aqueous solutions, as well as in 95% EtOH, can remain undetectable for weeks (O’Riordan et al. 2005). Therefore, we decided to apply acetone as a test solvent. Short acetone treatments can be expected to generate detectable differences between the sensor materials, as acetone is known to dissolve PtTFPP and to some extent PPFS (Paz-Pazos and Pugh 2005), but acetone does not immediately dissolve PS (Mark 1999).

**Fig. 7:**
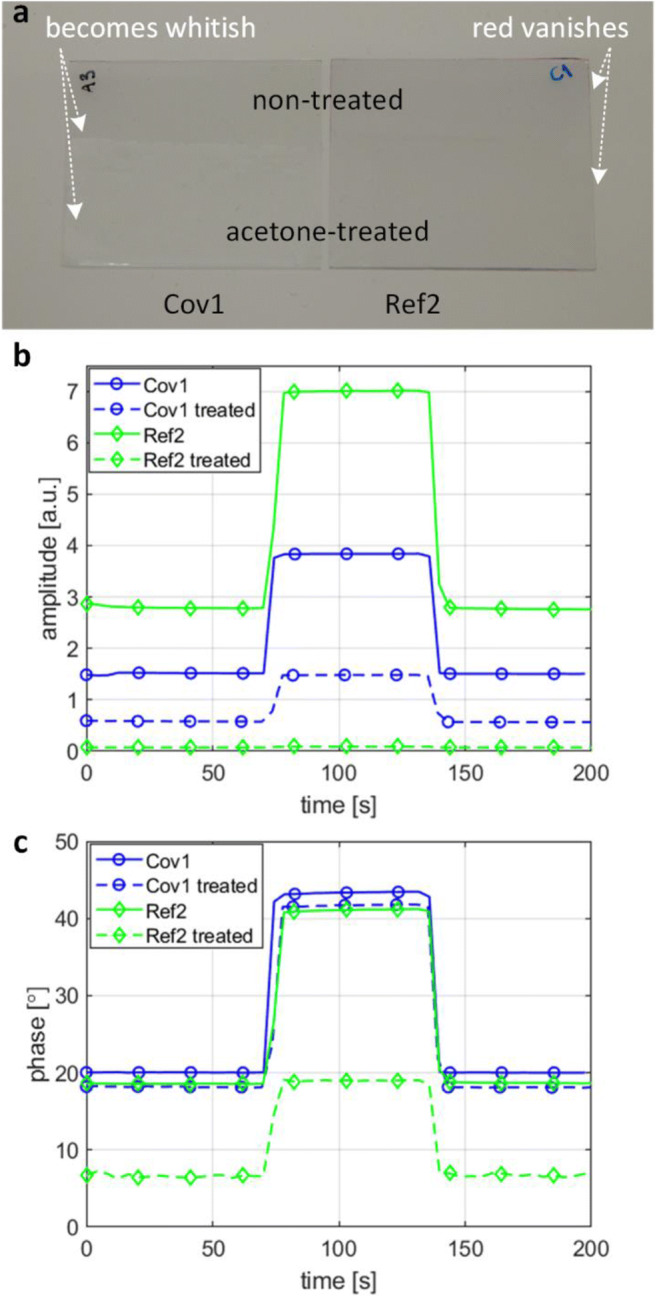
(**a**) Photographs of oxygen sensing films on the glass substrates after a partial acetone treatment. On the left, film with covalently immobilized PtTFPP (Cov1), and on the right, film with physically embedded PtTFPP (Ref2). The acetone-treated parts of the substrates have been immersed in acetone for 20 s. The corresponding oxygen sensing amplitude (**b**) and phase (**c**) responses on the non-treated sides (solid) and acetone-treated sides (dashed) at pO_2_ = 19 kPa and pO_2_ = 0 kPa

Glass plates with spin-coated films, containing either covalently immobilized (Cov1) or physically embedded (Ref2) PtTFPP, were immersed halfway in acetone for 20 s. The short acetone treatment typically turned film Cov1 into a less transparent, slightly white-colored film, probably due to a partial dissolution of PPFS (Fig. 7a). On the other hand, the mild red appearance in the Ref2 film typically vanished in the treatment, thus indicating that the most of the physically embedded PtTFPP has been leached out of the film. Figure 7b and c show the influence of the acetone treatment on the oxygen sensing performance. The amplitude responses (Fig. 7b) reveal that the emission of Ref2 has almost completely vanished after the treatment, thus indicating a strong leakage of PtTFPP. On the other hand, the same treatment only halves the emission power of Cov1, despite the decreased transparency of the film. The difference between the films is further illustrated in Fig. 7c, where the phase responses are shown. Due to the lost emission power, the noise in the phase signal of Ref2 is increased, and the absolute phase change is considerably reduced. However, the phase response of Cov1 is only slightly shifted, presumably due to the increased optical losses and modified oxygen diffusion rate. Indeed, the oxygen sensing performance of Cov1 remains almost the same. These results evidently suggest that the covalent immobilization substantially reduces the leaching of the indicators.

**Fig. 8:**
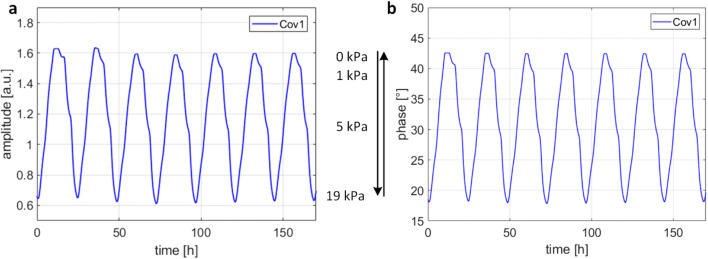
The luminescence amplitude (**a**) and phase (**b**) response in oxygen tension monitoring in deionized water. Sensing film with covalently immobilized indicators (Cov1) was used. The oxygen partial pressure was varied according to the sequence [19, 10, 5, 1, 5, 10, 19, 10, 5, 1, …] kPa

The oxygen sensing stability of the material Cov1 was further studied in a week-long experiment, where the calibration chamber was filled with DI water, and the oxygen partial pressure was changed stepwise between 19 kPa and 0 kPa. The luminescence responses were recorded four times a minute, and the results are shown in Fig. 8. The recorded responses demonstrate a solid luminescence intensity (Fig. 8a) and, in particular, luminescence lifetime stability (Fig. 8b). The minor fluctuations in the responses are due to changes in the ambient temperature.

## Conclusions

This paper studied the influence of the covalent immobilization of PtTFPP on various properties of the PS-based oxygen sensing material. As the main result, the material with covalently immobilized PtTFPP showed significantly reduced cytotoxic effects to the hiPSC-derived neurons and CMs, compared to reference materials with physically embedded PtTFPP or PtOEPK. In particular, the results with hiPSC-derived neurons were unequivocal, displaying a strong statistical significance. The results also suggest that the reduced cytotoxicity is due to the enhanced ability to limit the leaching of the indicators. Moreover, the experimental data, albeit small within this paper, suggests that phototoxicity is not on issue as long as the leaching of the indicators is prevented, and the excitation power radiated into the cell culture remains low.

There are relatively few reports on luminescence-based oxygen sensing with hiPSC-derived cultures, and the studies typically rely on commercial plates and devices (Kusuma et al. 2014; Abaci et al. 2010). However, it is challenging to integrate such devices into miniaturized systems, such as a modular cell culturing system with many simultaneous functionalities (Rajan et al. 2018). For such systems, the manufacturing method described in this paper can be applied when preparing PtTFPP-based oxygen sensing films, spots or beads. Moreover, this method may also be applied to host polymers other than PS.

## Electronic supplementary material

ESM 1 (DOCX 1340 kb)

## Data Availability

Available by request**.**
