# Supplementary material for: Covalent immobilization of luminescent oxygen indicators reduces cytotoxicity
Source: Biomed Microdevices. 2020 Jun 3;22(2):41. doi: 10.1007/s10544-020-00495-3 (PMC7270993; doi:10.1007/s10544-020-00495-3)
Supplement: Supplementary file 1 — (DOCX 1340 kb) [file 10544_2020_495_MOESM1_ESM.docx]

Supplementary material

for

Covalent immobilization of luminescent oxygen indicators reduces cytotoxicity

Hannu Välimäki^a,^^[[1]](#footnote-1)^, Tanja Hyvärinen^b^, Joni Leivo^a^, Haider Iftikhar^a^, Mari Pekkanen-Mattila^b^, Dhanesh Kattipparambil Rajan^a^, Jarmo Verho^a^, Joose Kreutzer^a^, Tomi Ryynänen^a^, Jonatan Pirhonen^b^, Katriina Aalto-Setälä^b^, Pasi Kallio^a^, Susanna Narkilahti^b^, Jukka Lekkala^a^

*^a^Faculty of Medicine and Health Technology, Tampere University, Korkeakoulunkatu 3, 33720 Tampere, Finland*

*^b^Faculty of Medicine and Health Technology, Tampere University, Arvo Ylpön katu 34, 33520 Tampere, Finland*


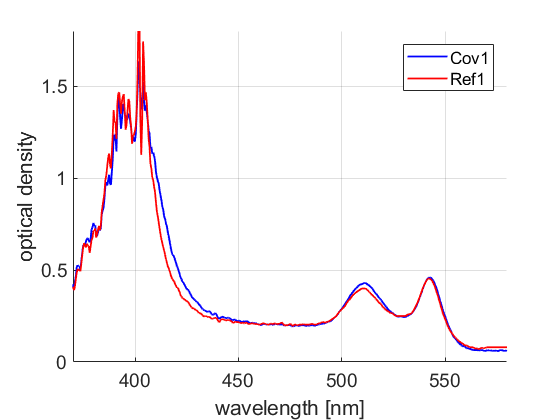


Fig. S1. The absorption spectra of the materials Cov1 and Ref1 dissolved in toluene (4.0 wt-%). Material Ref1 contains 0.05% of PtTFPP.

Fig. S2. The normalized absorption (A) and emission (B) spectra of the solid materials Cov1 and Ref1 on glass substrate.

**Cytotoxicity test with hiPSC-derived CMs**

Oxygen sensing spots were manufactured on the bottom of 18 cell wells of individual sensing materials (Cov1 × 10, Ref1 × 4, Ref3 × 4). Six wells without sensing spots were used as a basic reference (glass controls, GC). The CMs were plated and followed for up to 13 days in an incubator. However, the size of the data set decreased significantly after the first week of the test, when the optical evaluation revealed that the sensor spots had disappeared from four wells, probably during the medium change that took place on day five. The decrease in the size of the data set necessitated reorganization of the data. In the end, the statistical analysis was performed on day 13 (D13) with the following three data sets: i) Cov1 (9 wells); ii) all materials with physically embedded indicators combined into a single reference set denoted as Ref (1 × Ref1 wells and 4 × Ref3 wells); (iii) control (6 wells) (see Table S2 in Online Resource 1).

Fig. S3 shows representative phase contrast images of the CM clusters in separate wells on D13. In Fig. S3a, a poorly attached (and non-beating) cluster is located next to the sensing spot made of the material containing physically embedded PtOEPK (Ref3), and a close-up of the same cluster is shown in Fig. S3b. The cluster differs morphologically from the cluster in the control well, shown in Fig. S3d. On the other hand, the well attached (and strongly beating) cluster in Fig. S3c, located very close to the sensing spot made of the material with covalently immobilized indicators (Cov1), resembles morphologically the cluster in the control well (Fig. S3d). In addition, many clusters close to the Cov1 material were partly attached and spread over the spot itself; thus, indicating very low or non-existing cytotoxicity of the sensing material Cov1 (Fig. S3c).

Fig. S3e summarizes the attachment and beating score values on D13. The values demonstrate the superiority of the material with covalently immobilized indicators. Indeed, the difference between the Cov1 and Ref is evident, while the difference between Cov1 and GC is small or non-existent. These facts are supported by the Mann-Whitney test showing a statistically significant difference between Cov1 and Ref (p < 0.05), but not between Cov1 and Ref.

**
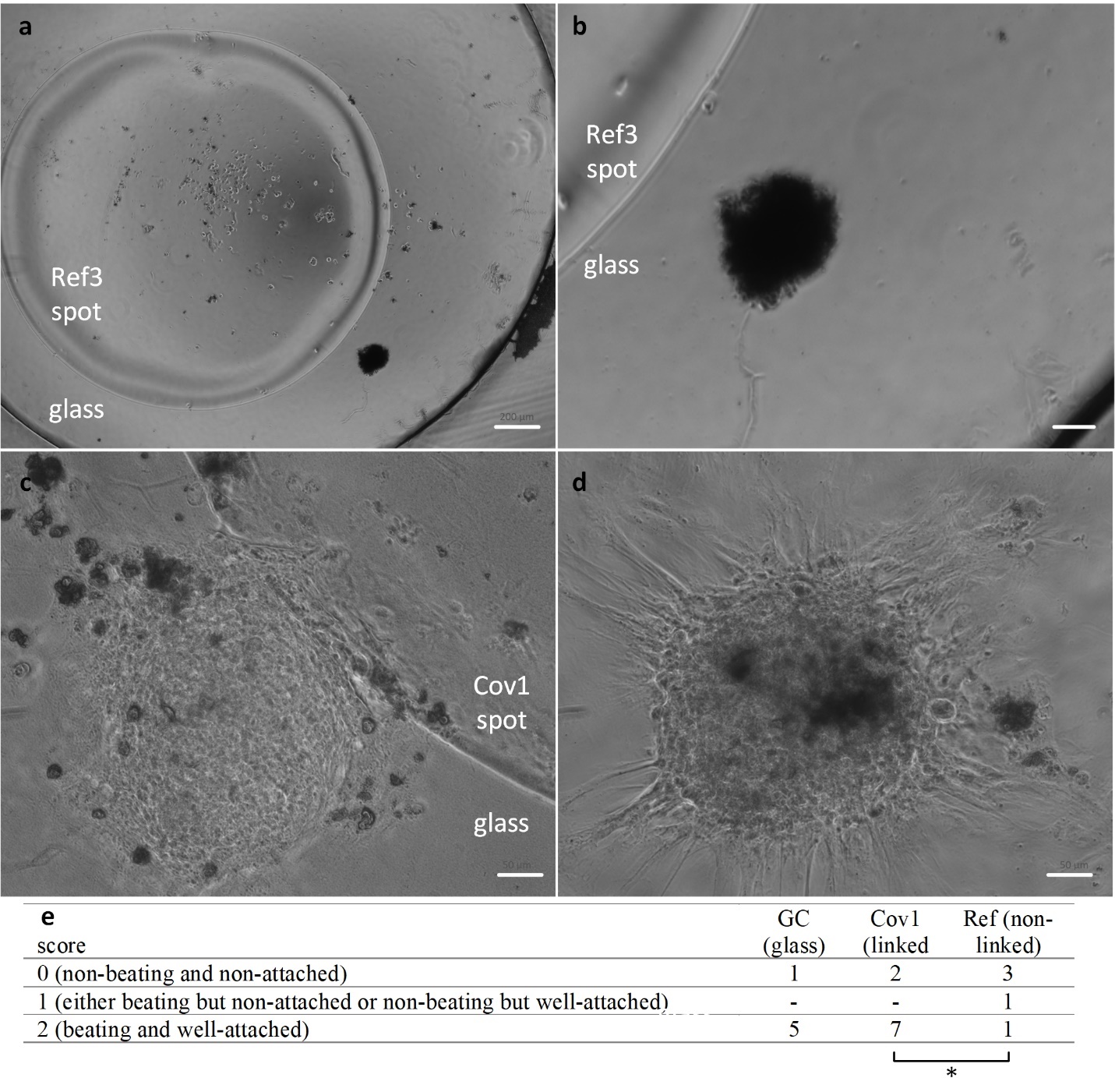
Fig.** **S3** Representative phase contrast images and the classification of the cultured hiPSC-derived cardiomyocyte clusters on day 13. (a) Score 0; a non-beating and non-attached cluster next to a sensing spot with physically embedded PtOEPK (Ref3). (b) A close-up of the cluster in (a). (c) Score 2; a well-attached and strongly beating cluster in the edge of the spot with covalently immobilized PtTFPP (Cov1). (d) Score 2; a well-attached and strongly beating cluster on the control well (GC). Scale bar 200 µm in (a) and 50 µm (b, c, d). (e) A summary of the scores. Statistical analysis according to Mann-Whitney U-test (two-tailed). Statistical significances are denoted as * p < 0.05

Table S1. Number of samples in live/dead and immunocytochemical staining of neuronal cells.

|  | **Live/dead staining** | | **Immunocytochemical staining** | |
| --- | --- | --- | --- | --- |
|  | Experiment 1 | Experiment 2 | Experiment 1 | Experiment 2 |
| **PS control** | - | 18 | - | 18 |
| **Cov1** | 10 | 27 | 10 | 27 |
| **Ref1** | 9 | 18 | 9 | 18 |
| **Ref2** | - | 18 | - | 17 |
| **Ref3** | 9 | - | 10 | - |

Table S2. Number of samples in viability assessment of cardiomyocyte clusters.

|  | **Viability** | |
| --- | --- | --- |
|  | Experiment 1 | After 5 days |
| **GC** | 6 | 6 |
| **Cov1** | 10 | 9 |
| **Ref1** | 4 | 1 |
| **Ref2** | - |  |
| **Ref3** | 4 | 4 |

1. Corresponding author. Tel. +358405511695. ORCID 0000-0002-9611-1711. Email: hannu.valimaki@tuni.fi. [↑](#footnote-ref-1)
